# Increase in the end-tidal carbon dioxide concentration during mediastinoscopic esophagectomy: a comment to a report of Haraguchi et al.

**DOI:** 10.1186/s40981-025-00837-1

**Published:** 2026-01-07

**Authors:** Katsuhide Masui, Tomoyuki Saito, Takero Arai, Takashi Asai

**Affiliations:** Department of Anesthesiology, Dokkyo Medical University Saitama Medical Center, 2-1- 50 Minami-Koshigaya, Koshigaya, Saitama 343- 8555 Japan

**Keywords:** Mediastinoscopy, Tracheal injry, EtCO2

To the Editor:

We read with interest a report of Dr. Haraguchi and colleagues, of a case of tracheal injury diagnosed by a sudden increase in end-tidal carbon dioxide (EtCO₂) during mediastinoscopic esophagectomy [1]. We experienced a similar case and describe a cautionary tale.

Written informed consent was obtained from the patient for publication. A 83-yr- old man, 160 cm, 56 kg, was scheduled for mediastinoscopic esophagectomy. In the operating room, after insertion of a thoracic epidural catheter, general anesthesia was induced with propofol 60 mg, remifentanil 0.5 µg·kg⁻¹·min⁻¹, and rocuronium 40 mg was injected intravenously. A tracheal tube (NIM TriVantage™ EMG Endotracheal Tube; Medtronic, Tokyo, Japan) was inserted without difficultly. Anesthesia was maintained with sevoflurane, fentanyl, and remifentanil. The lungs were ventilated with pressure-controlled mode (inspiratory pressure: 15 cmH_2_O, respiratory rate: 8/min, a fraction of inspired oxygen (FiO_2_): 0.47. The tidal volume was approximately 450 mL and the EtCO_2_ was within the normal limits.

Approximately 13 min after the start of insufflation of CO_2_ to the mediastinum, the EtCO_2_ suddenly increased to 125 mmHg, with no other abnormal vital signs except for a gradual decrease in the tidal volume to 400 mL. Arterial pressure of CO_2_ (PaCO₂) was 83 mmHg and of oxygen (PaO_2_) 155 mmHg.

Mediastinoscopy was temporarily stopped, after which EtCO₂ rapidly decreased to the normal range. As there was no apparent abnormality and the vital signs were stable, mediastinoscopy was restarted, but the EtCO₂ immediately increased again to 125 mmHg. Mediastinoscopy was stopped, which led EtCO_2_ to decrease to the normal range. We speculated that a high concentration of CO_2_ was inadvertently insufflated from mediastinum to the tracheal lumen. Bronchoscopy confirmed a laceration at the membranous portion of the trachea near the carina. The tracheal injury was repaired under mediastinoscopy. At the end of surgery, the tracheal tube was removed, and the patient was transferred to the ward without complications.

If the trachea is injured, CO_2_ may be in inadvertently insufflated to the airway, and if the amount of CO_2_ insufflated inadvertently is large, there is a potential risk of death by reduced concentrations of oxygen and by toxic effects of CO_2_ [2]. In our case, the EtCO_2_ increased to 125 mmHg, indicating at least 16% of end-tidal gas was CO_2_. In the report of Haraguchi and colleagues [1], the EtCO_2_ increased to 227 mmHg (approximately 30%CO_2_), which was associated with hypotension and bradycardia.

We have learned from the manufacture of the device (Multi-Gas/Flow Unit, Nihon Kohden, Tokyo, Japan) used that 125 mmHg is the upper limit value of the partial pressure for CO_2_, and 99–150 mmHg for the other capnographs currently available. We do not know which capnograph (which indicated 227 mmHg) Dr. Haraguchi and colleagues used, but the capnographs in general cannot detect critically high CO_2_ concentrations in the respiratory system. This may lead to unexpected hypoxemia and, in the worst case, cardiac arrest.

In conclusion, we should be aware that capnographs cannot detect a life-threatening increase of CO_2_ in the respiratory system during mediastinoscopic esophagectomy.

## Data Availability

Not applicable.
